# Development and assessment of a lysophospholipid-based deep learning model to discriminate geographical origins of white rice

**DOI:** 10.1038/s41598-017-08892-0

**Published:** 2017-08-17

**Authors:** Nguyen Phuoc Long, Dong Kyu Lim, Changyeun Mo, Giyoung Kim, Sung Won Kwon

**Affiliations:** 1 Research Institute of Pharmaceutical Sciences and College of Pharmacy, Seoul National University, Seoul, 08826 Republic of Korea; 2 National Institute of Agricultural Sciences, Rural Development Administration, Jeonju, 54875 Republic of Korea; 3 Plant Genomics and Breeding Institute, Seoul National University, Seoul, 08826 Republic of Korea

## Abstract

Geographical origin determination of white rice has become the major issue of food industry. However, there is still lack of a high-throughput method for rapidly and reproducibly differentiating the geographical origins of commercial white rice. In this study, we developed a method that employed lipidomics and deep learning to discriminate white rice from Korea to China. A total of 126 white rice of 30 cultivars from different regions were utilized for the method development and validation. By using direct infusion-mass spectrometry-based targeted lipidomics, 17 lysoglycerophospholipids were simultaneously characterized within minutes per sample. Unsupervised data exploration showed a noticeable overlap of white rice between two countries. In addition, lysophosphatidylcholines (lysoPCs) were prominent in white rice from Korea while lysophosphatidylethanolamines (lysoPEs) were enriched in white rice from China. A deep learning prediction model was built using 2014 white rice and validated using two different batches of 2015 white rice. The model accurately discriminated white rice from two countries. Among 10 selected predictors, lysoPC(18:2), lysoPC(14:0), and lysoPE(16:0) were the three most important features. Random forest and gradient boosting machine models also worked well in this circumstance. In conclusion, this study provides an architecture for high-throughput classification of white rice from different geographical origins.

## Introduction

The abiotic stress has a large impact on the constituents of plant sources, such as food additives, pharmaceuticals, flavors, and industrially important biochemicals^1^. In recent years, the demand for high-quality food products with geographical indications has substantially increased^2^. Adulteration practice, especially the falsification of food origins, is prejudicial to consumers as well as authorized producers and distributors^2, 3^. Therefore, the geographical origin determination and authenticity of food products have become the major issues of food industry. White rice, a main staple food of many countries in Asia and Africa, has been a potential target to adulteration regarding their similar physical properties^4^. Better authentication methods to detect the geographical origin are, indeed, required.

Trace elements and stable isotope ratios have been widely used to discriminate the geographical origins of rice^5–8^. When search for other potential chemical compositions that are capable to predict the geographical origins of commercial white rice, we found that phospholipids (PLs) are the attractive targets. Environmental factors, which are essentially different from countries to countries, greatly affect the concentrations of PLs in white rice. In addition, the deterioration of some PL species during storage contributes to the degradation of white rice^9^. In a previous preliminary experiment, we demonstrated that the differences of lysoglycerophospholipids (lysoGPLs) might be proper to differentiate white rice originated from different countries^10^.

There are many analytical methods for the determination of white rice geographical origins based on their chemical compositions^2^. In addition, chemometric-based classification techniques, especially partial least squares discriminant analysis (PLS-DA), have been formally applied for the authenticity of food products and herbal medicines, including white rice^11–14^. Interestingly, a recent survey provided a background about the statistical methods the researchers have used in metabolomics-related studies^15^. Univariate statistic has been a common practice, especially Student t-test (91%) and analysis of variance (89%). Other methods include Mann–Whitney U test (54%), Benjamini–Hochberg false discovery rate correction (50%), and Kruskal Wallis (44%). In multivariate analysis, principal component analysis (PCA) (96%) and PLS-DA (73%) are the two most widely used methods. However, random forest (RF) was employed in only 27%. It is worth mentioning that overoptimistic and overfitting results are the common problems of the PLS-DA and the abovementioned methods, except RF, are not the preferred options for the classification study^16^. Besides these well-known statistical and chemometric methods, the application of sophisticated machine learning techniques in the geographical classification has also emerged in recent years^17^. Supervised machine learning algorithms are very powerful and they can additionally be applied to get better insights into the alteration patterns of the biological targets under specific conditions^18^. Maione *et al*. successfully employed machine learning to classify the origins of rice of different regions within a country^19^. The experiment was executed using 20 trace elements and the origins of the samples was predicted by support vector machines, RF, and neural network^20–22^. The applied models were validated using repeated 10-time 10-fold cross-validation. Although the sample size was relatively small and there was no independent validation sample, the results demonstrated the great potential of the supervised learning techniques in geographical classification of white rice. Additionally, deep learning is an advanced machine learning approach and has recently become the cutting-edge algorithm because of its extraordinary performance of the prediction accuracy in many fields^23–28^. The good profile and advancement of deep learning encourage us to utilize this approach for the geographical classification of commercial white rice.

In the current paper, we developed a method for rapid, accurate, and reproducible discrimination of the geographical origins of white rice from different countries. Since the generalization of the results is crucially important in class prediction study, we have collected a large number of white rice samples belonging to 30 different cultivars (11 from Korea and 19 from China). In addition, white rice cultivated in two different years, 2014 and 2015, were collected in three different time points. Sixty representative samples of white rice cultivated from 2014 was collected in 2015. White rice cultivated from 2015 was collected in April (40 representative samples) and August 2016 (26 representative samples). Moreover, our recent developed method for simultaneous profiling of 17 prominant lysoGPLs in white rice using direct infusion-electrospray ionization-multiple reaction monitoring-mass spectrometry (DI-ESI-MRM-MS) was applied in this study^10^. This significantly reduced the time required to analyze data for the classification down to few minutes compared to the conventional chromatography coupled with MS methods. lysoGPL data were further processed, visualized, and analyzed using a wide range of techniques for data exploration and machine learning-based classification. Finally, the proposed prediction model from white rice cultivated in 2014 was implemented to predict the origins of the samples from two different batches of white rice cultivated in 2015. Our results indicate that the combination of DI-MRM-MS-based targeted lipidomics with the cutting-edge deep learning algorithm provides an effective framework for the authenticity and geographical origin determination of white rice.

## Results and Discussion

### Summary of 2014 white rice, 2015-early white rice and 2015-late white rice

A total of 126 samples belonging to 30 different cultivars were purchased in April-2015 (2014 white rice, batch 1), April-2016 (2015 white rice, batch 2), and August-2016 (2015 white rice, batch 3) at local markets. There were 60, 40, and 26 samples in batch 1, batch 2, and batch 3, respectively. The detailed information can be found in Table 1.

**Table 1:** The geographical origins and the cultivars of white rice from Korea and China.

Group	Korea			China		
	Label^*^	Origin	Cultivar	Label^*^	Origin	Cultivar
**Batch 1** *Training set*	KR1	Gyeonggi	Choochung	CN1	Heilongjiang	Jinjingdao
	KR2	Gyeonggi	Samgwang	CN2	Heilongjiang	Youjida
	KR3	Gangwon	Ode	CN3	Liaoning	Dongbeida
	KR4	Jeonnam	Hopyeong	CN4	Shandong	Dongbeida
	KR5	Jeonnam	Ode	CN5	Heilongjiang	Wuchangxiang
	KR6	Jeonbuk	Shindongjin	CN6	Liaoning	Zhenzhu
	KR7	Jeonnam	Ode	CN7	Jilin	Daohuaxiang
	KR8	Gangwon	Ode	CN8	Liaoning	Daohuaxiang
	KR9	Jeonnam	Ilmi	CN9	Heilongjiang	Zhanglixiang
	KR10	Jeonnam	Ode	CN10	Jilin	Baijinxiang
	KR11	Gyeongbuk	Ilmi	CN11	Liaoning	Zhenzhu
	KR12	Jeonnam	Samgwang	CN12	Heilongjiang	Fuxiangdao
	KR13	Chungnam	Samgwang	CN13	Liaoning	Yalujiang 7 xi
	KR14	Gyeongbuk	Ilpum	CN14	Jilin	Youjida
	KR15	Chungnam	Samgwang	CN15	Liaoning	Yalujiang 3 xi
	KR16	Gyeongbuk	Senoori	CN16	Shandong	Dongbeida
	KR17	Gyeonggi	Choochung	CN17	Heilongjiang	Zhanglixiang
	KR18	Gangwon	Ode	CN18	Heilongjiang	Yatian
	KR19	Gyeonggi	Choochung	CN19	Heilongjiang	Xuejingdao
	KR20	Gangwon	Choochung	CN20	Heilongjiang	Zhonghuahe
	KR21	Jeonbuk	Shindongjin	CN21	Heilongjiang	Wuchangda
	KR22	Gyeongnam	Samgwang	CN22	Jilin	Youjida
	KR23	Chungbuk	Choochung	CN23	Jilin	Daohuaxiang
	KR24	Jeonnam	Hitomebore	CN24	Shandong	Zhanglixiang
	KR25	Jeonnam	Ilmi	CN25	Heilongjiang	Youjida
	KR26	Gyeonggi	Samgwang	CN26	Liaoning	Daohuaxiang
	KR27	Gyeonggi	Koshihikari	CN27	Heilongjiang	Shengtaidao
	KR28	Gyeongbuk	Ilmi	CN28	Jilin	Luseda
	KR29	Gyeonggi	Shindongjin	CN29	Liaoning	Dongbeida
	KR30	Gyeonggi	Jinsang	CN30	Heilongjiang	Yueguangdaoxi
**Batch 2** *Test set 1*	KR1	Chungnam	Samgwang	CN1	Jilin	Youjida
	KR2	Gyeongbuk	Ilpum	CN2	Heilongjiang	Shengtaidao
	KR3	Gyeonggi	Shindongjin	CN3	Heilongjiang	Yatian
	KR4	Gyeongbuk	Ilmi	CN4	Jilin	Luseda
	KR5	Gangwon	Choochung	CN5	Jilin	Daohuaxiang
	KR6	Jeonnam	Ode	CN6	Heilongjiang	Fuxiangdao
	KR7	Gyeonggi	Choochung	CN7	Liaoning	Daohuaxiang
	KR8	Gyeongbuk	Ilmi	CN8	Heilongjiang	Xuejingdao
	KR9	Gangwon	Ode	CN9	Liaoning	Zhenzhu
	KR10	Gyeonggi	Choochung	CN10	Jilin	Daohuaxiang
	KR11	Gyeongnam	Samgwang	CN11	Jilin	Youjida
	KR12	Jeonnam	Ilmi	CN12	Shandong	Zhanglixiang
	KR13	Gangwon	Ode	CN13	Heilongjiang	Zhanglixiang
	KR14	Gyeonggi	Jinsang	CN14	Liaoning	Zhenzhu
	KR15	Jeonnam	Hopyeong	CN15	Liaoning	Yalujiang 7 xi
	KR16	Jeonnam	Hitomebore	CN16	Heilongjiang	Wuchangda
	KR17	Chungnam	Samgwang	CN17	Liaoning	Dongbeida
	KR18	Chungbuk	Choochung	CN18	Heilongjiang	Zhanglixiang
	KR19	Jeonbuk	Shindongjin	CN19	Liaoning	Dongbeida
	KR20	Gyeonggi	Koshihikari	CN20	Jilin	Baijinxiang
**Batch 3** *Test set 2*	KR1	Gyeonggi	Jinsang	CN1	Heilongjiang	Fuxiangdao
	KR2	Gyeonggi	Choochung	CN2	Liaoning	Yalujiang 7 xi
	KR3	Gyeonggi	Koshihikari	CN3	Heilongjiang	Dongbeida
	KR4	Gyeongnam	Samgwang	CN4	Jilin	Luseda
	KR5	Chungnam	Samgwang	CN5	Liaoning	Shengtai
	KR6	Jeonbuk	Shindongjin	CN6	Jilin	Baijinxiang
	KR7	Gangwon	Ode	CN7	Heilongjiang	Daohuaxiang
	KR8	Gangwon	Ode	CN8	Heilongjiang	Fuxiangdao
	KR9	Gyeonggi	Choochung	CN9	Jilin	Baijinxiang
	KR10	Jeonbuk	Shindongjin	CN10	Heilongjiang	Dongbeida
	KR11	Gyeonggi	Choochung	CN11	Jilin	Yatian
	KR12	Gyeongbuk	Samgwang	CN12	Heilongjiang	Daohuaxiang
	KR13	Jeonnam	Ilmi	CN13	Liaoning	Zhenzhu

Some cultivars were purchased at the same province. However, they were cultivated in different areas and processed by different companies.

In general, the geographical classification of white rice from different countries is difficult because there are many factors such as water, temperature, light, ion, nutrient, and reactive oxygen species that greatly affect the reproducibility of the results^29^. The cultivation and harvest time (within-year or different years), the diversity of white rice cultivars (genetically modified or not), and storage conditions are also particularly significant. From the practice aspect, the influence of the quality of the sample preparation and data gathering methods are remarkable. In this study, we developed an experimental design that aimed to partially overcome the abovementioned difficulties and to achieve the results with generalization. Indeed, we collected white rice that was cultivated in different years (2014 white rice and 2015 white rice), white rice that was cultivated in the same year but the farming season and storage period were different (early 2015 white rice and late 2015 white rice). The sample collection was performed with the intention to maximize the heterogeneity of the samples by sampling many cultivars or white rice with different within-country origins. Finally, it is also worth pointing out that lysoGPLs profiling of white rice were conducted in three different periods.

### Characterization of lysoGPLs in white rice

Although the quantity of PLs is much lower than other compounds in white rice, nutritional impact of PLs has been regconized^30^. Furthermore, lysoGPLs, a member of PLs, has an important role in determining rice quality. lysoPCs and lysoPEs are two major types of lysoGPLs in white rice and lysoPEs are particularly vulnerable to environmental changes. lysoPGs, however, just occupy a very small quantity in rice endosperm^9^. The existent of other lysoGPLs such as lysophosphatidylinositol (lysoPIs), lysophosphatidylserine (lysoPSs), and lysophosphatidic acid (lysoPAs) are as-yet unknown. Our investigation aimed to characterize six classes of lysoGPLs in commercial white rice, including lysoPCs, lysoPEs, lysoPGs, lysoPIs, lysoPSs, and lysoPAs. However, only 17 lysoGPLs of lysoPC (6 species), lysoPE (7 species), and lysoPG (4 species) were capable to be detected^10^. Moreover, the divergence of the lysoGPLs in white rice samples originating from different countries was described. The study implemented DI-MRM-MS, which substantially reduces the quantity of samples and the analysis time yet yields valuable data. Therefore, 17 lysoGPLs were initially profiled in this study in search for an effective classification model to discriminate white rice between Korea and China.

### lysoGPLs variation of white rice from different countries: data exploration and visualization

**Figure 1:**
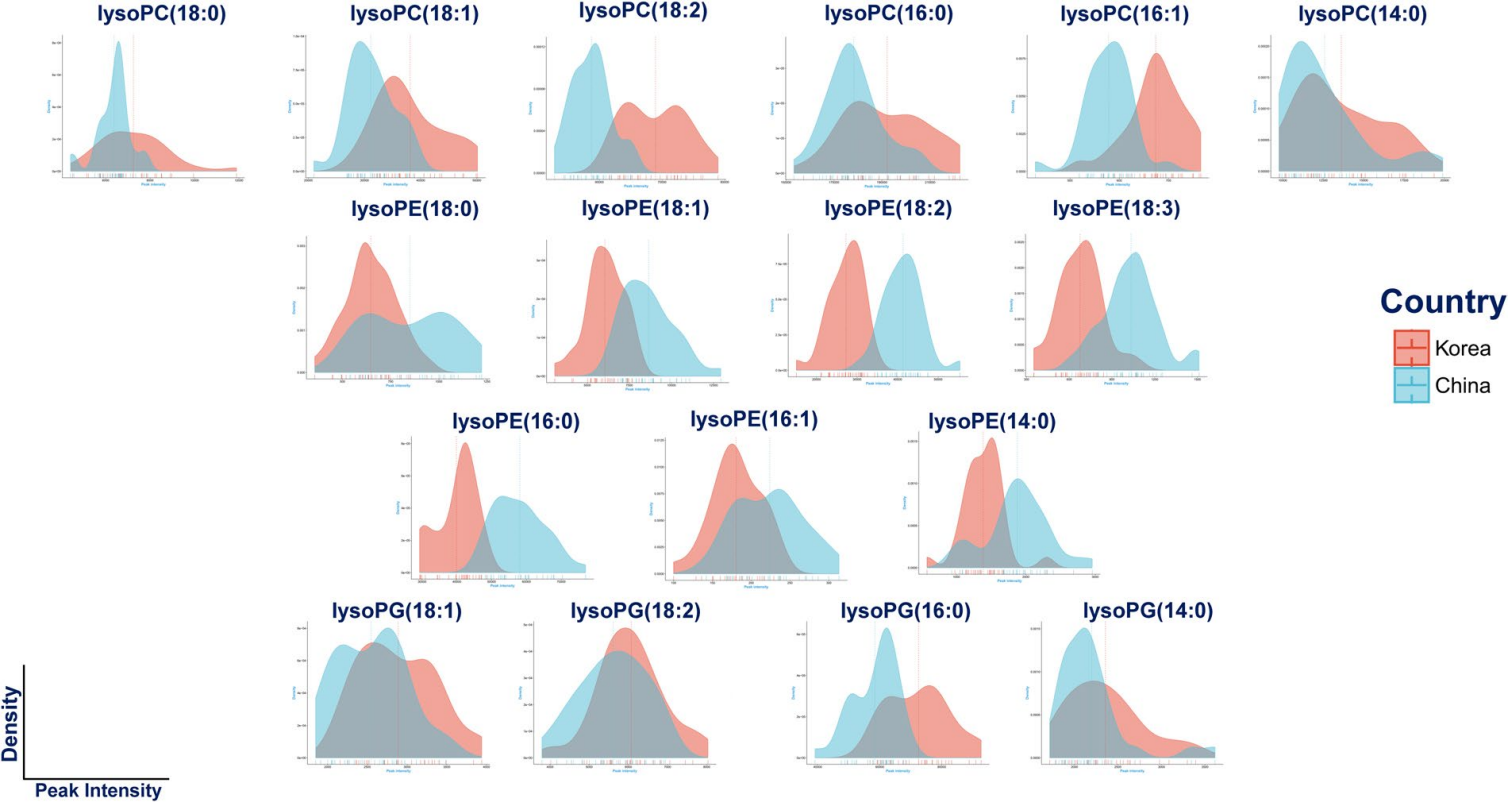
Density plots of 17 lysGPLs of 2014 white rice from Korea and China. lysoPCs are enriched in white rice from Korea while lysoPEs are prominent in white rice from China.

The density plots in Fig. 1 show the distribution of the intensities of 17 lysoGPLs in white rice originated from Korea and China of batch 1. The density plots of two batches of 2015 white rice are provided in Figure S1. In general, the relative differences in terms of the concentrations of 17 species among samples between two countries were small. Among three batches of samples, the concentrations of lysoPCs were higher in white rice from Korea. In contrary, the concentrations of lysoPEs were elevated in white rice from China. lysoPGs were likely enriched in Korean group, however, the results were not consistent. The fold change, *P*-value, and FDR of 17 lysoGPLs among three batches of samples can be found in Table 2. In 2014 white rice, the concentrations of 14 species were statistically significant differences, except lysoPC(14:0) and lysoPG(14:0), and lysoPG(18:2). Similarly, the concentrations of 12 species were statistically significant differences, except lysoPC(16:1), lysoPE(14:0), lysoPG(14:0), lysoPG(18:1), and lysoPG(18:2) in 2015-early white rice. Finally, the concentrations of 13 species were statistically significant differences, except lysoPC(16:0), lysoPC(16:1), lysoPC(18:2), and lysoPE(18:0), in 2015-late white rice. Noticeably, the values of fold changes were relative small and there was no big difference between two groups (with the criterion of 2). Collectively, these results suggested a slight deviation in terms of the lysoGPLs concentrations of white rice and this is likely results from the heterogeneity of many affecting factors, such as cultivation year and storage conditions.

**Table 2:** The fold change, *P*-value, and FDR of 17 lysoGPLs among three different batches of samples.

Group	Compound	Fold change (K/C)	*P*-value	FDR
Batch 1	lysoPC(14:0)	1.08	8.78E-2	8.78E-2
	lysoPC(16:0)	1.08	1.44E-3	2.04E-3
	lysoPC(16:1)	1.17	8.68E-8	2.46E-7
	lysoPC(18:0)	1.14	1.91E-2	2.32E-2
	lysoPC(18:1)	1.22	2.91E-6	6.19E-6
	lysoPC(18:2)	1.17	2.49E-13	1.41E-12
	lysoPE(14:0)	0.74	1.17E-5	2.20E-5
	lysoPE(16:0)	0.69	1.18E-16	2.01E-15
	lysoPE(16:1)	0.81	1.58E-4	2.68E-4
	lysoPE(18:0)	0.76	9.26E-4	1.43E-3
	lysoPE(18:1)	0.70	3.09E-10	1.31E-9
	lysoPE(18:2)	0.66	3.21E-16	2.73E-15
	lysoPE(18:3)	0.65	1.43E-8	4.86E-8
	lysoPG(14:0)	1.07	8.10E-2	8.61E-2
	lysoPG(16:0)	1.24	4.22E-7	1.02E-6
	lysoPG(18:1)	1.13	8.68E-3	1.13E-2
	lysoPG(18:2)	1.08	6.68E-2	7.56E-2
Batch 2	lysoPC(14:0)	1.50	2.32E-8	9.85E-8
	lysoPC(16:0)	1.09	2.88E-6	5.45E-6
	lysoPC(16:1)	1.08	4.82E-1	5.12E-1
	lysoPC(18:0)	1.27	4.07E-7	1.10E-6
	lysoPC(18:1)	1.30	7.37E-9	4.18E-8
	lysoPC(18:2)	1.17	2.02E-9	1.71E-8
	lysoPE(14:0)	0.91	2.91E-1	3.30E-1
	lysoPE(16:0)	0.71	4.53E-7	1.10E-6
	lysoPE(16:1)	0.89	4.52E-2	6.41E-2
	lysoPE(18:0)	0.79	3.75E-4	6.37E-4
	lysoPE(18:1)	0.75	9.25E-7	1.97E-6
	lysoPE(18:2)	0.69	1.45E-11	2.47E-10
	lysoPE(18:3)	0.71	2.96E-7	1.01E-6
	lysoPG(14:0)	1.09	6.34E-2	8.30E-2
	lysoPG(16:0)	1.08	1.23E-2	1.91E-2
	lysoPG(18:1)	0.98	6.65E-1	6.65E-1
	lysoPG(18:2)	0.93	7.18E-2	8.72E-2
Batch 3	lysoPC(14:0)	1.13	1.02E-2	1.44E-2
	lysoPC(16:0)	1.07	8.11E-2	9.20E-2
	lysoPC(16:1)	1.04	4.88E-1	5.19E-1
	lysoPC(18:0)	1.38	2.67E-5	9.09E-5
	lysoPC(18:1)	1.23	1.30E-4	3.69E-4
	lysoPC(18:2)	1.00	9.60E-1	9.60E-1
	lysoPE(14:0)	0.62	4.93E-4	1.05E-3
	lysoPE(16:0)	0.66	1.92E-7	1.09E-6
	lysoPE(16:1)	0.79	1.96E-2	2.56E-2
	lysoPE(18:0)	0.86	7.26E-2	8.82E-2
	lysoPE(18:1)	0.78	6.28E-4	1.19E-3
	lysoPE(18:2)	0.63	1.92E-7	1.09E-6
	lysoPE(18:3)	0.62	1.25E-3	2.08E-3
	lysoPG(14:0)	1.31	1.35E-3	2.08E-3
	lysoPG(16:0)	1.52	1.92E-7	1.09E-6
	lysoPG(18:1)	1.55	5.77E-6	2.45E-5
	lysoPG(18:2)	1.32	4.93E-4	1.05E-3

**Figure 2:**
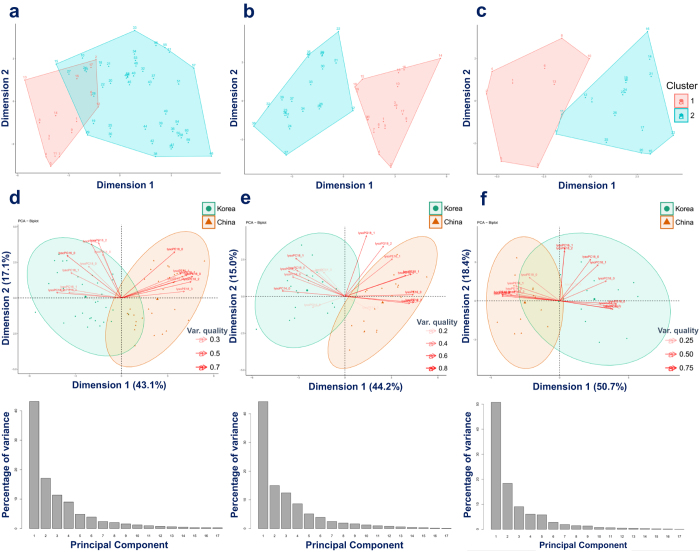
PAM and PCA analyses for data exploration. **(a–c)** Show two clusters of PAM of 2014 white rice, 2015-early white rice, and 2015-late white rice, respectively. **(d**–**f)** Show PCA biplots of 2014 white rice, 2015-early white rice, and 2015-late white rice, respectively. (**a**) 1–30: white rice from Korea, 31–60: white rice from China. (**b**) 1–20: white rice from Korea, 21:40: white rice from China. **(c)** 1–13: white rice from Korea, 14–26: white rice from China.

**Figure 3:**
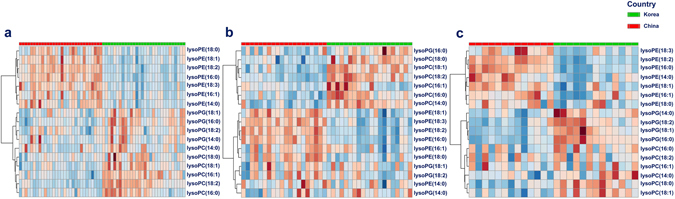
Heatmaps show the relative difference of concentrations of 17 lysoGPLs of **(a)** 2014 white rice, **(b)** 2015-early white rice, and **(c)** 2015-late white rice, respectively.

Univariate analysis does not consider the correlations among features, thus, we further conducted unsupervised multivariate exploratory data analyses to get better insights into our data sets^31^. PAM cluster analysis was first applied to observe the dissimilarity of the samples of three data sets. This algorithm is preffered because it is robust to outliers^32^. Unexpectedly, many samples that belonged to Korean group were clustered together with Chinese group (Fig. 2a) in 2014 white rice. In other two batches of samples from 2015 white rice, this unsupervised analysis showed a similar clustered tendency, however, with a lower degree since some samples of Korean group were clustered together with the samples from Chinese group (Fig. 2b and c). PCA, a data reduction unsupervised method, was conducted to explore the patterns of difference between white rice from Korea and China. As shown in Fig. 2d, a partly overlap (95% confident interval (CI)) between two groups was observed (PC1 + PC2 = 60.2%). Significantly, lysoPCs were shown to be important in Korean group while lysoPEs were prominent in Chinese group. Similar trends were also observed in two batches of 2015 white rice (Fig. 2e and f). Heatmap was also applied to get the intuitive visualization of our data sets. As shown in Fig. 3, the stronger colors focused on the lysoPEs and lysoPCs of Chinese groups and Korean groups, respectively. In general, there was no feature with unusually extremely colors in the three data sets. Collectively, the univariate analysis and multivariate unsupervised data exploration revealed that there was an overlap in some degree of white rice originated from two countries and cultivated in different years. The observation also implied that the geographical classification of white rice might be difficult for conventional methods. Consequenly, sophicated classification algorithms are more proper for this task.

### Development and validation of white rice geographical classification

Highly correlated variables, which include lysoPG(14:0), lysoPE(18:1), lysoPC(18:1), lysoPE(18:0), lysoPG(18:2), lysoPE(16:1), and lysoPG(18:1) were removed from the data sets. The correlation matrix can be seen in Figure S2. The 10 remaining predictors with a two-class label of 2014 white rice data set was finally used to train the deep learning model for geographical classification of white rice. The model was trained with an input layer, four hidden layers (200 neurons/layer), and an output layer. The iteration (epochs) of 10 was set. A five-fold cross-validation was applied to estimate the prediction performance of the model in the training set. We used the adaptive learning rate algorithm, as recommended by H2O. There are several regularization method options. Among them, dropout is currently the method of choice to prevent overfitting^33^. When select dropout regularization, random neurons in hidden layers will be excluded during the training process to prohibit the dependencies that might occur^34^. Thus, the rectified activation function with dropout (the dropout ratio – 0.5) was selected in this study. Early stopping was applied with the stopping metric – log loss, stopping tolerance – 0.001, and stopping rounds – 5. The variable importance was extracted from the prediction model. A seed number was set to get the reproducible results. Other parameters were kept as default.

**Figure 4:**
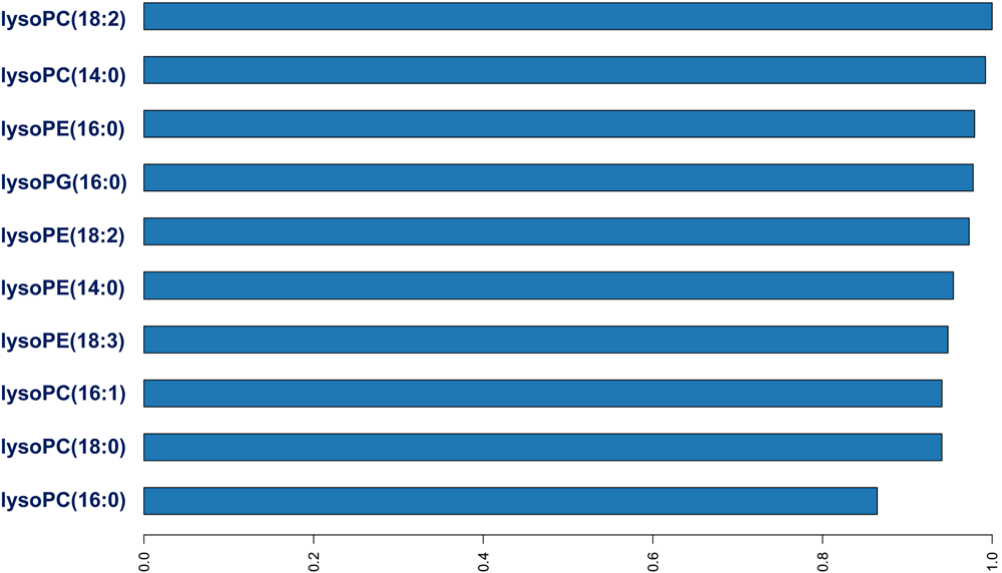
Variable importance plot of the optimal deep learning model. Top three predictors are lysoPC(18:2), lysoPC(14:0), and lysoPE(16:0).

The trained prediction model was then applied to predict the class of unseen samples from two batches of 2015 white rice. The two batches are different in terms of the collection time (April and August 2016). The results were surprisingly encouraging. As shown in Table 3, the RMSE and log loss values of three different classification analyses were small. For instane, the RMSE of the training set, test set 1, and test set 2 were 0.45, 0.54, and 0.46, respectively. Similarly, log los values of the training set, test set 1, and test set 2 were 0.55, 0.83, and 0.59, respectively. There was no class error so the MCE of classification analyses was 0 in three data sets. Furthermore, AUC, Gini, accuracy, sen, spec, TPR, and TNR were as the highest level (1.00). Look at the variable importance (Fig. 4), 10 predictors contribute significantly to the deep learning model. However, lysoPC(16:0) tended to be the least important predictor. The top three predictors were lysoPC(18:2), lysoPC(14:0), and lysoPE(16:0). Last, but not least, we were aware of the architecture of the above settings, which might be more complicated than needed. For example, the number of the layers could be decreased down to two, each with 200 neurons. Of note, we are free to tune the model using the training set as long as the tuned model is capable to predict the origins of the samples correctly. Nevertheless, the act of “training on the test set” should be avoided. The three data sets and corresponding R commands for deep learning classification are provided in Spreadsheet S1.

**Table 3:** The performance of the deep learning prediction model on training and test sets.

Data set	Total samples	RMSE	log loss	MCE	AUC	Gini	Accuracy	Sensitivity	Specitivity	TPV	TNV
White rice 2014 (Training set)	60	0.45	0.55	0.00	1.00	1.00	1.00	1.00	1.00	1.00	1.00
White rice 2015 (A) (Test set 1)	40	0.54	0.83	0.00	1.00	1.00	1.00	1.00	1.00	1.00	1.00
White rice 2015 (B) (Test set 2)	26	0.46	0.59	0.00	1.00	1.00	1.00	1.00	1.00	1.00	1.00

**RMSE:** Root mean squared error.

**LogLoss:** Logarithmic loss.

**MCE:** Mean per-class error.

**AUC**: Area under the ROC curve.

**Gini:** Gini coefficient.

**TPR:** True positive rate.

**TNR:** True negative rate.

Next, we examined the geographical classification of the RF model with the settings of followings: the number of tree in the forest (ntrees) – 1000, five-fold cross-validation, and other parameters were set at default. In addition, the parameters of the GBM for geographical classification were: ntrees – 100, five-fold cross-validation, learn rate – 0.1, stopping metric – log loss, stopping round – 5, stopping tolerance – 0.0001, score tree interval – 10. Other parameters were set as default. The results of both RF and GBM were convincing since there was only one sample from Chinese group (RF) and one sample from Korean group (GBM) of the 2015 white rice of the test set 1 were misclassified. The information of RMSE, log loss, MCE, AUC, Gini, and variable importance of the RF and GBM optimal models can be found in Figure S3. In the RF model, lysoPE(18:2), lysoPE(16:0), and lysoPC(18:2) turned out to be the top three important predictors whilst the role of lysoPC(18:0), lysoPC(14:0), and lysoPC(16:0) were insignificant. However, in the GBM model, lysoPE(18:2) and lysoPE(16:0) were the two most important features and the role of others appeared to be negligible.

Our study has several limitations. First, the sample size was relatively small due to the practical reasons. This might increase the overfitting of the classification models. However, we applied dropout and early stopping as well as external validation method using within-year and between-year samples to guarantee the regularization of the results. The sample size issue may also be solved when new white rice samples are available in the market. Second, the intended mixing ingradients of the samples between two countries were not investigated. Finally, the scope of the study was limitted to commercial white rice of Korea and China. Further investigations, therefore, are warranted to extend the utility of this approach to the real-world applications.

## Conclusion

lysoGPLs can be considered as the potential features for geographical authenticity of white rice. In fact, our findings demonstrate the combination of simultaneous lysoGPL profiling method and advanced supervised learning algorithms can effectively predict the origins of the white rice. In addition to deep learning, random forest and gradient boosting machine techniques have proven to be the probable methods. In conclusion, this study suggests that machine learning algorithms possibly improve the geographical discrimination of white rice as well as other food products. Owing to the great potential of this approach, prospective studies are needed to broaden its application to a larger scale either in the coverage of geographical origins or the geographical authencity of other food products.

## Materials and Methods

### Materials and reagents

One hundred twenty-six white rice samples were randomly collected from local markets in Korea and China. After collection, the samples were immediately stored at −70 °C until further processed. The solvents (analytical grade), including methanol, acetonitrile, and isopropanol, were purchased from J. T. Baker (Avantor, Phillipsburg, NJ, USA). Caffeine was obtained from Sigma-Aldrich (St Louis, MO, USA). Polytetrafluoroethylene (PTFE) syringe filter (0.20 µm) was purchased from Advantec (Tokyo, Japan).

### Sample preparation

White rice was freeze-dried and finely grinded to powder. The powder was then strained using two sieves with different sizes (250 µm and 125 µm) and extracted using a previously described protocol^30^. Concisely, 1 mg caffeine was added to 150 mg of powder samples. The mixture was extracted using 6 mL of 75% isopropanol in a water bath at 90 °C for 2 h and centrifuged at 16,000 g for 5 min. Thereafter, 1 mL of supernatant filtered by a PTFE syringe filter was transferred to a Agilent 1.5 mL screw vial (Agilent, CA, USA) for the analysis.

### DI-MRM-MS analysis conditions

A triple-quadrupole mass spectrometry system (6460 QqQ LC-ESI-MS/MS, Agilent, CA, USA) was exploit to perform every experiment in order to ascertain the practical instrumental conditions. The following settings were adopted from our previously developed method^10^. The analysis of lysoPCs was conducted in positive ion mode. lysoPEs and lysoPGs, on the other hand, were characterized in negative ion mode. The contamination of ion source by sample injection was minimized using a constant flow of 50% acetonitrile (0.2 mL/min). The sample sequences of every experiment were set randomly to avoid possible technical bias. The mass spectrometer was following the acquisition settings: scan time −200 scans/sec, cell accelerator voltage −7 V, fragmentor voltage −135 V, nebulizer pressure −40 psi, dry gas temperature −325 °C, dry gas flow −11 L/min, and capillary −4 kV. Nitrogen was used as the collision, nebulizing, and drying gas. The system was operated at a collision energy of 20 eV for positive and negative ion modes. MRM transitions of each compound were set in accordance to the mass per charge ratios (*m/z*) of the highest intensity fragments of product ions. The experiment was tightly controlled and a variation criterion of 10% of relative standard deviations (RSD) in quality control (QC) samples was used to consider the quality of the analysis of targeted lipid species. Lastly, the lipid identification was confirmed using our in-house library.

### Data preprocessing and univariate statistical analysis

DI-MRM-MS data were processed using Agilent Mass Hunter Workstation software version B.06.00. The peak intensities of 17 lysGPLs were normalized using peak intensities of caffeine. There were no near zero-variance and missing values in the three data sets. Density plots were used to visualize the intensity distributions of samples between two countries and Wilcoxon rank-sum test was performed to detect differentially expressed features. A *P*-value of <0.05 and a false discovery rate (FDR) for multiple testing of <0.1 were considered to be the level of statistical significance. The univariate analysis was performed using Metaboanalyst 3.0 and the density plot was illustrated using ggpubr 0.1.2 in R language 3.3.3^35–37^.

### Data visualization and multivariate Data Analysis

Since multivariate analysis does take the correlations among variables into account, it is considered particularly suitable for analyzing high-dimensional omics data^31^. In this study, partitioning around medoids clustering analysis (PAM), PCA, and heatmap analysis were applied to visualize the data and explore the tendency of separation among samples. Except heatmap analysis that was performed using metaboanalyst 3.0, other analysis and visualization techniques were performed using FactoMineR version 1.35, factoextra 1.0.4, and ggplot 2 2.2.1 in R language version 3.3.3^37–40^.

### Highly correlated predictor removal

Highly correlated predictors might affect the performance of the prediction models. Therefore, we removed all the predictors with absolute correlations of 0.70 or higher. The process was conducted using caret package 6.0–73. Correlation matrix was visualized using corrplot 0.77 package^41, 42^.

### Deep learning classification

In this study, a feedforward deep neural network model for class prediction was established using 60 white rice samples cultivated in 2014. A five-fold cross-validation was utilized during training process as a model validation technique. The performance of the model was further validated using two independent batches of white rice cultivated in 2015. The training and testing processes were carried out using H2O package 3.10.3.6 in R language version 3.3.3. H2O provides cutting-edge machine learning algorithms and well-known regularization tools for big data analysis^43^. Although deep learning includes unsupervised and supervised settings, H2O provides a purely supervised learning protocol together with many innovative features that help getting the optimal prediction models in a short period. In addition, RF and gradient boosting machine (GBM), two major machine learning techniques, were additionally employed to build classification models^37, 44, 45^. The metrics to evaluate the model included root mean squared error (RMSE), cross-entropy loss function (log loss), mean per-class error (MCE), the area under the receiver operating characteristic (ROC) curve (AUC), and Gini coefficient (Gini) along with the prediction accuracy, sensitivity (sen), specificity (spec), true positive value (TPV), and true negative value (TNV).

## Electronic supplementary material

Supplementary Figure1-3

Full data and R commands
