# Supplementary material for: Development and assessment of a lysophospholipid-based deep learning model to discriminate geographical origins of white rice
Source: Sci Rep. 2017 Aug 17;7:8552. doi: 10.1038/s41598-017-08892-0 (PMC5561257; doi:10.1038/s41598-017-08892-0)
Supplement: Supplementary file 1 — Supplementary Figure1-3 [file 41598_2017_8892_MOESM1_ESM.pdf]

# <Supporting materials>

## The development and assessment of a lysophospholipid-based deep learning model to discriminate geographical origins of white rice

Nguyen Phuoc Long[1,#], Dong Kyu Lim[1,#], Changyeun Mo[2], Giyoung Kim[2], Sung Won Kwon*[,1,3]

[1] Research Institute of Pharmaceutical Sciences and College of Pharmacy, Seoul National University, Seoul 08826, Republic of Korea

[2] National Institute of Agricultural Sciences, Rural Development Administration, Jeonju 54875, Republic of Korea

[3] Plant Genomics and Breeding Institute, Seoul National University, Seoul 08826, Republic of Korea

[#]: these authors contributed equally to this work

***Corresponding author:** Sung Won Kwon. Email address: swkwon@snu.ac.kr

**<Figure S1> Density plots of 2015-early white rice, and 2015-late white rice.**

Density plots of 17 lysGPLs of white rice from Korea and China (early 2015 white rice)

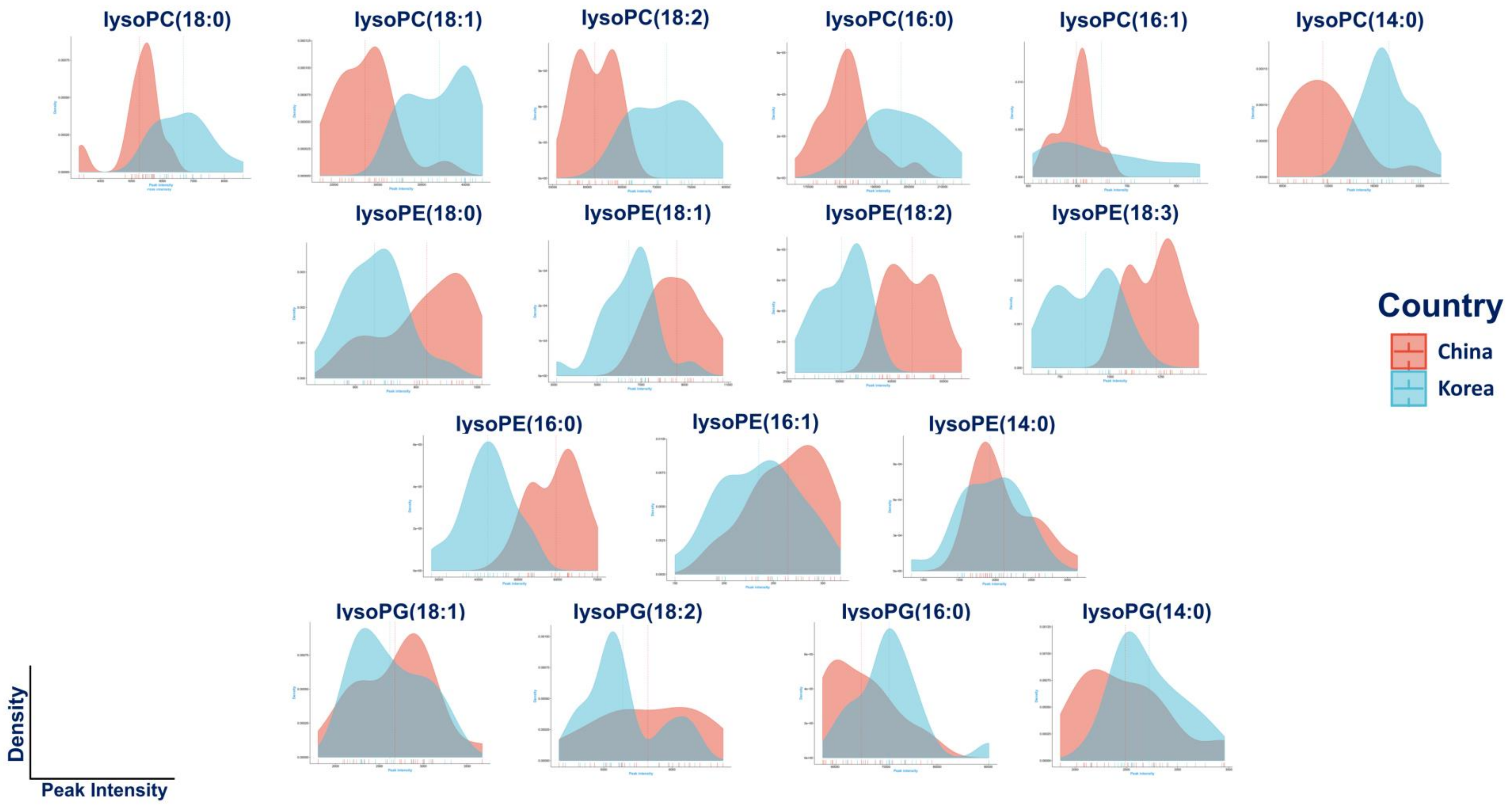

Density plots of 17 lysGPLs of white rice from Korea and China (late 2015 white rice)

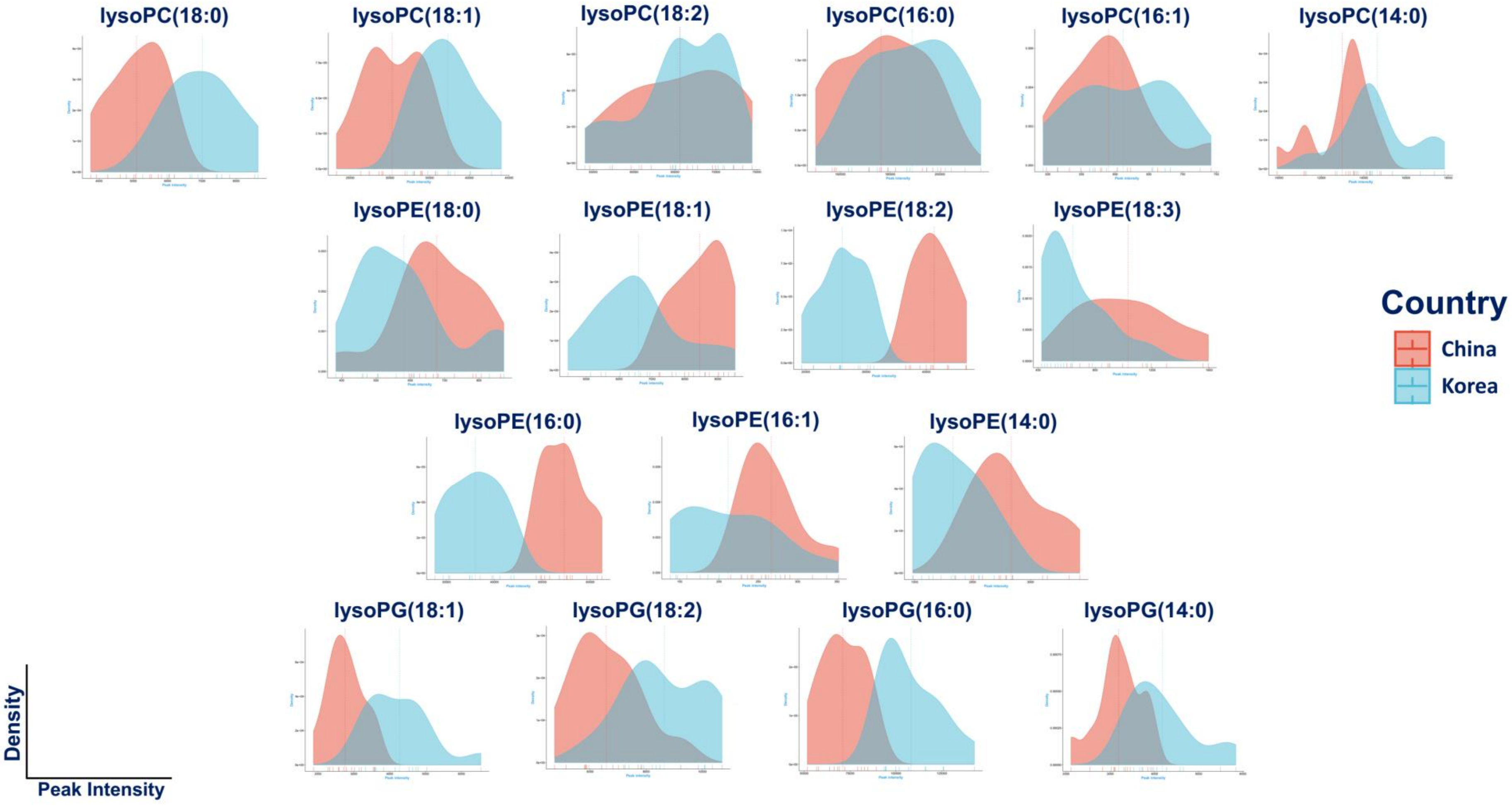

**<Figure S2> The matrix of highly correlated variables of 2014 white rice.**

Highly correlated variables matrix of 2014 white rice from the correlation analysis using Caret package. Eventually, lysoPG(14:0), lysoPE(18:1), lysoPC(18:1), lysoPE(18:0), lysoPG(18:2), lysoPE(16:1), and lysoPG(18:1) were removed from the final data sets.

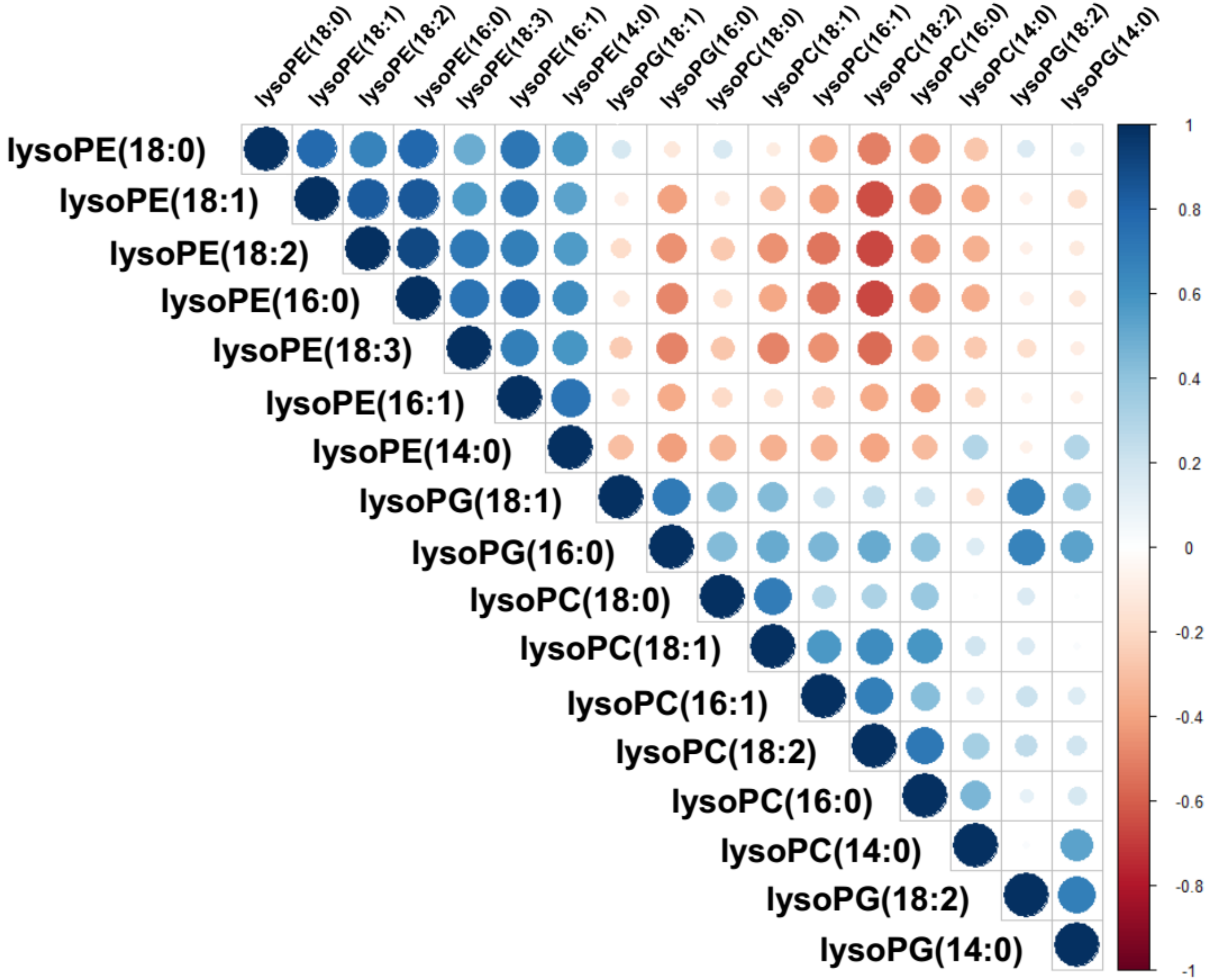

**<Figure S3> The classification performance and variable importance of random forest and gradient boosting machine.**

The classification performance of random forest model

| Data set | Total samples | RMSE | log loss | MCE | AUC | Gini |
|---|---|---|---|---|---|---|
| White rice 2014 (Training set) | 60 | 0.11 | 0 | 0.00 | 1.00 | 1.00 |
| White rice 2015 (A) (Test set 1) | 40 | 0.25 | 0.21 | 0.03 | 0.99 | 0.99 |
| White rice 2015 (B) (Test set 2) | 26 | 0.14 | 0.12 | 0.00 | 1.00 | 1.00 |

**RMSE:** Root mean squared error
**Log loss:** Logarithmic loss
**MCE:** Mean per-class error
**AUC:** Area under the ROC curve
**Gini:** Gini coefficient

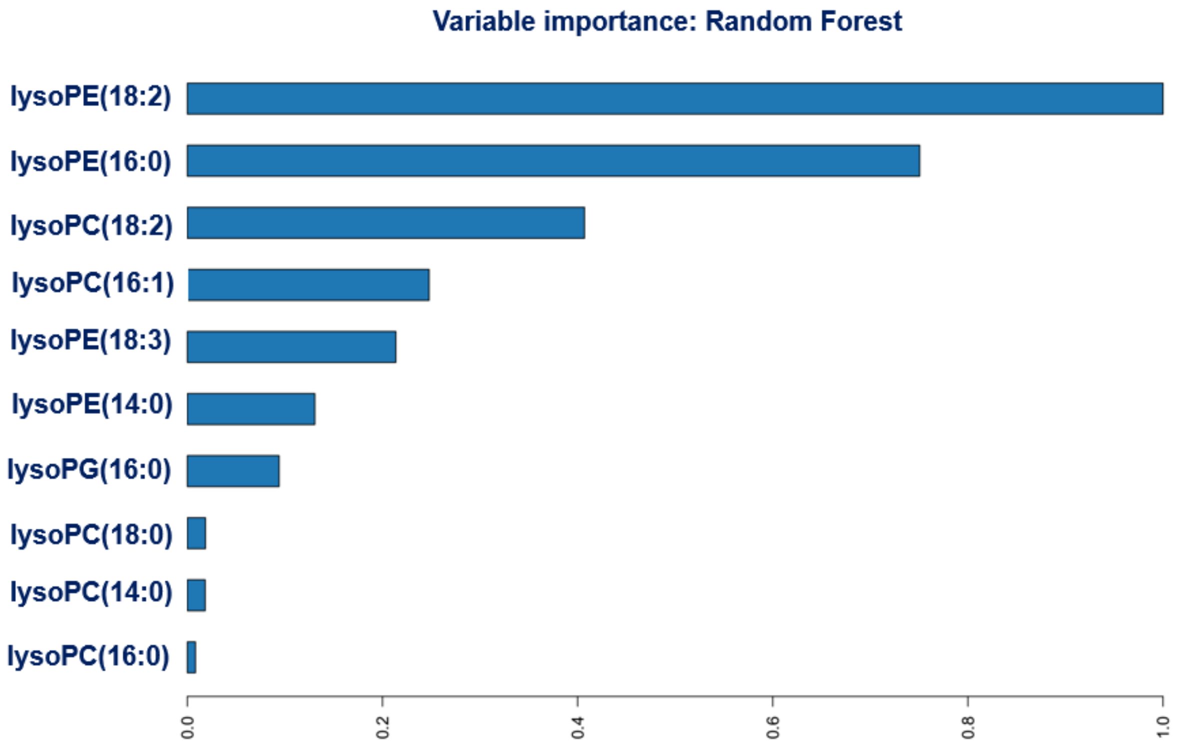

The classification performance of gradient boosting machine model

| Data set | Total samples | RMSE | log loss | MCE | AUC | Gini |
|---|---|---|---|---|---|---|
| White rice 2014 (Training set) | 60 | 3.20E-4 | 1.94E-4 | 0.00 | 1.00 | 1.00 |
| White rice 2015 (A) (Test set 1) | 40 | 0.28 | 0.41 | 0.03 | 0.99 | 0.98 |
| White rice 2015 (B) (Test set 2) | 26 | 2.34E-4 | 1.57E-4 | 0.00 | 1.00 | 1.00 |

**RMSE:** Root mean squared error
**Log loss:** Logarithmic loss
**MCE:** Mean per-class error
**AUC:** Area under the ROC curve
**Gini:** Gini coefficient

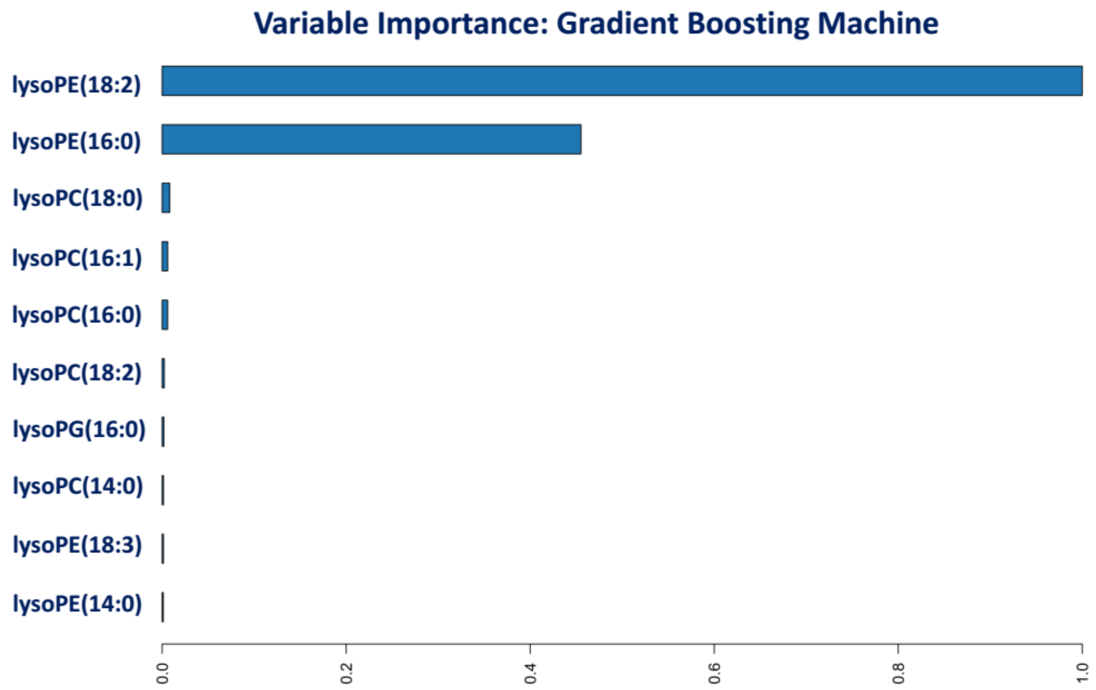

**<Spreadsheet S1> Raw data and corresponding R commands of deep learning classification**

Please refer to excel file (Spreadsheet S1. Supporting data-Full-data-and-R-commands.xlsx)
